# Optimization of Abrasive Water Jet Machining of SiC Reinforced Aluminum Alloy Based Metal Matrix Composites Using Taguchi–DEAR Technique

**DOI:** 10.3390/ma14216250

**Published:** 2021-10-20

**Authors:** Muthuramalingam Thangaraj, Mahmoud Ahmadein, Naser A. Alsaleh, Ammar H. Elsheikh

**Affiliations:** 1 Department of Mechatronics Engineering, SRM Institute of Science and Technology, SRM Nagar, Kattankulathur, Chennai 603203, India; 2 Mechanical Engineering Department, Imam Mohammad Ibn Saud Islamic University, Riyadh 11564, Saudi Arabia; m.ahmadein@f-eng.tanta.edu.eg (M.A.); naalsaleh@imamu.edu.sa (N.A.A.); 3 Department of Production Engineering and Mechanical Design, Faculty of Engineering, Tanta University, Tanta 31527, Egypt

**Keywords:** aluminum alloy, SiC, DEAR, optimization, AWJM

## Abstract

Since the importance of introducing new engineering materials is increasing, the need for machining such higher strength materials has also considerably increased. In the present research, an endeavor was made to introduce a Taguchi–DEAR methodology for the abrasive water-jet machining process, while machining a SiC-reinforced aluminum composite. Material removal rate, taper angle, and surface roughness were considered as the quality measures. The optimal arrangement of input process factors in the AWJM process was found to be 2800 bar (WP), 400 mg/min (AF), 1000 mm/min (FR), and 4 mm (SOD), among the chosen factors, with an error accuracy of 0.8%. The gas pressure had the most significance for formulating the performance measures, owing to its ability to modify the impact energy and crater size of the machined specimen.

## 1. Introduction

Currently, considerable attention is given to finding new engineering composites with higher strength and lower density, for various manufacturing applications. Aluminum composites are the most used engineering materials, because of their distinct physical and mechanical properties, such as low density, high load carrying capacity, and high elastic modulus [1]. Aluminum alloys are reinforced with ceramic particles such as SiC, TiB_2_, and B_4_C to synthesize metal matrix composites (MMC) with enhanced mechanical properties [2]. MMC materials are often subjected to post-processing machining and forming processes, in order to obtain final engineering products [3]. It is tedious to machine such higher strength MMCs using conventional machining processes. Hence, unconventional machining process, such as electro-chemical machining (ECM), electro-discharge machining (EDM), laser-beam machining (LBM), and abrasive-water-jet machining (AWJM) are the best choice to cut these kinds of materials [4].Due to its low production rate and high machining time, the EDM process is not favorable for cutting MMC materials [5]. The ECM process has the drawback of producing a corrosive layer on the machined surface [6]. While, the LBM process produces a recast layer and a wide heat-affected zone (HAZ) [7]. Hence, AWJM was proposed to avoid these drawbacks and to enhance the quality of the product using different performance measures [8].The AWJM process is an unconventional machining process, in which high-pressurized water mixed with high-strength abrasive particles is utilized for the cutting process. When the water stream contacts the specimen surface, material is removed owing to the mechanical impact energy. It is essential to optimize the process control factors during the machining process, due to of its non-linear nature [9]. The machining process can be evaluated using multiple performance measures. Therefore, the conventional Taguchi technique, which depends on optimizing a single response, needs to be employed with multi-response optimization techniques (MCDT), such as the weight assignment method, artificial neural-networks (ANN), response-surface methodology (RSM), and Taguchi–Grey relational analysis (TGRA) [10].A good understanding is needed to analyze the data for interpretation with the RSM approach [11]. Weight assignment is a straightforward method, which is very easy to implement. However, its prediction accuracy is limited. TGRA involves the tedious and complex step of choosing the Grey co-efficient, according to the ability of the process control factors [12]. The ANN approach provides better accuracy of performance prediction. However, it involves complex computational procedures and the integration of optimization algorithms, which entail a high computational cost [13]. Hence, Taguchi–data-envelopment-analysis-based-ranking (DEAR) is proposed in the current study, owing to its simplicity and considerable accuracy [14].

Although many research works are available on the machining of ceramic reinforced aluminum composites, only a few studies are available examining the influence of process control factors on quality measures during the cutting of this kind of composite using an AWJM process. Only a few research works have tried to adopt Taguchi-DEAR as a MCDT method in the AWJM process. Hence, this experimental investigation was carried out to optimize the AWJM cutting of a difficult to cut composite material, consisting of an aluminum matrix reinforced by SiC particles, and to achieve the following goals:

⮚ Employing a Taguchi–DEAR method as a new MCDT tool to obtain an optimal arrangement of process control factors of the machining process.
⮚ Analyzing the influence of process control factors in the AWJM process using different performance measures.
⮚ To identify the most significance factors effecting the performance measures.

## 2. Experimental Methodology

### 2.1. Synthesization of Al7075 Matrix Composite Reinforced by SiC Particles

**Figure 1:**
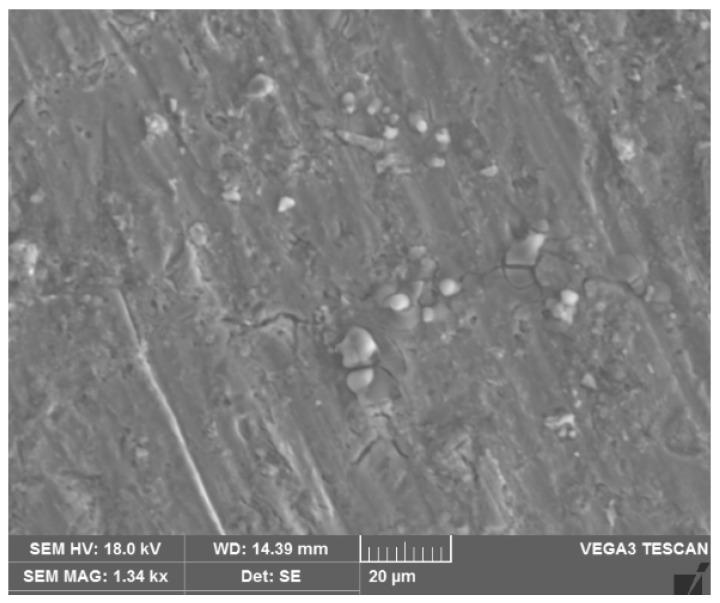
Surface characterization of the synthesized composite using SEM.

Aluminum (Al7075) alloy was utilized as the matrix alloy, owing to its distinctive properties and use in aerospace and automobile industries. SiC particles with an average size of 45 microns were utilized as the reinforcement, at under 10% weight, since they can produce a homogeneous distribution in the matrix alloy. In a stir casting approach, the matrix alloy was loaded in a crucible made up of graphite. Then it was heated using an electrical furnace to 650 °C. The micron sized SiC particles were preheated to 450 °C to remove the oxide film and then mixed with the matrix alloy. Then stirring was performed with a stainless steel impeller at the speed of 600 rpm to ensure a homogeneous distribution of particles. The stirring was carried out using a drilling chuck for about 45 min, under a constant stirring speed, within the furnace itself. The optimal diameter of the stirrer is the size at which the solid particles are fluidized in both the central and marginal parts at the same speed. Then, the stirred mixture was poured into a prepared mold with the required geometry. The mold was cooled down gradually to room temperature. Figure 1 shows a scanning electron microscope (SEM: JEOL JAPAN, Jeol-6490 JED-2300) image of the prepared Al7075 aluminum matrix reinforced by SiC particles. The distribution of the reinforcement particles was observed for the matrix alloy, with a uniform distribution based on a top view of the machined surface. The machining zone can also be analyzed using a SEM image based on the ASME B46.1-1995 standard, which specifies a magnification of 2000 in the vertical direction and 50 in the horizontal direction for the machining zone.

### 2.2. AWJM Cutting of Al7075 Matrix Composite Reinforced by SiC Particles

**Figure 2:**
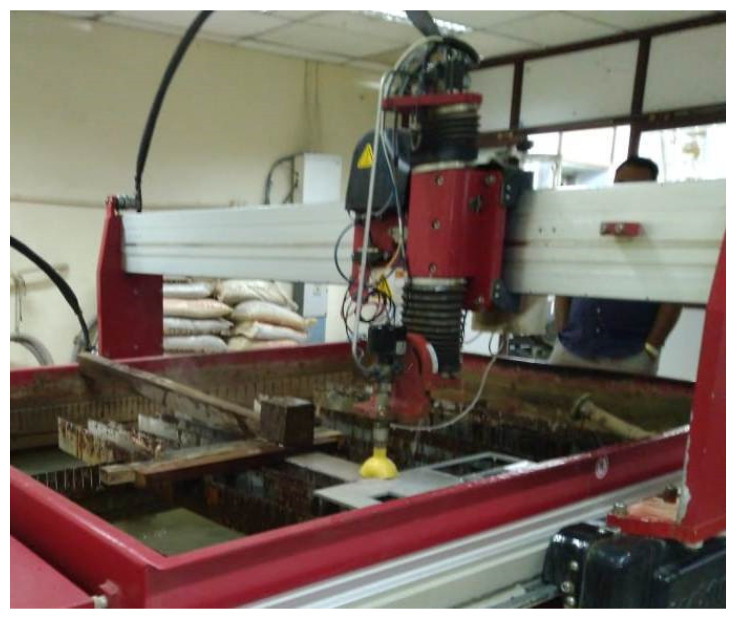
OMAX MAXIEM 1515 AWJM arrangement used in the present study.

The cutting investigations were carried out using an OMAX MAXIEM 1515 AWJM system at the speed of 12,700 mm/min and using a nozzle radius of 0.38 mm, as shown in Figure 2. A constant abrasive flow rate of 0.432 Kg/min was used during the cutting process. Owing to the importance of the control factors in the AWJM process, the abrasive particle flow rate (AF), water pressure (WP), standoff distance (SD), and feed rate (FR) were taken as the input process factors in the current work. Composite workpiece samples of 5-mm thickness were utilized for the AWJM based cutting. Since the experiments were carried out under low, moderate, and high level ratings of impact energy, the process control variables were decided as depicted in Table 1. The type of abrasive particles used in the cutting process plays a noteworthy role in the overall performance of the AWJM process. Therefore, garnet with a mesh size of 40 in were used as abrasive particles the current study.

**Figure 3:**
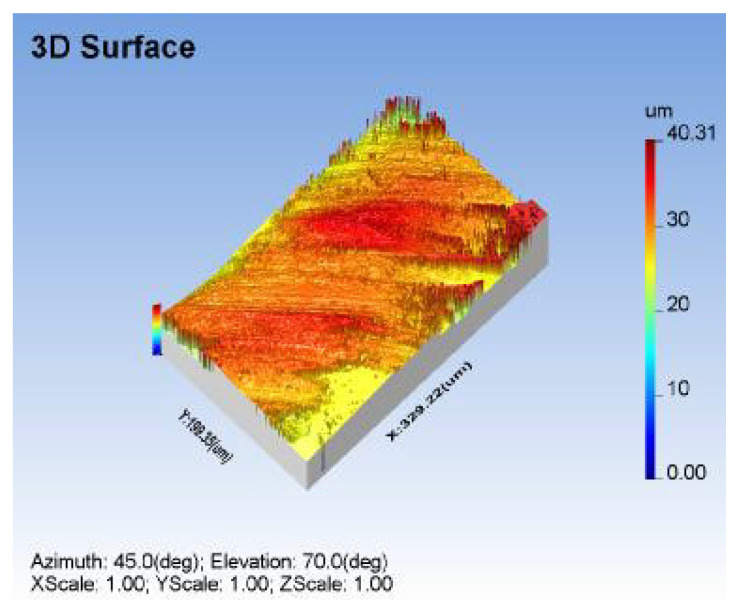
Measurement of R_a_ and surface texture under Trial 9.

Due to their ability to decide the performance measures, material removal rate (MRR), taper angle (TA), and surface-roughness (R_a_) were chosen as the output factors in the current investigation. The MRR was determined by computing the difference between the weight of the machined workpiece and the as-received one. This can be denoted by mm^3^/min. The R_a_ of the machined specimen was evaluated using anon-surface roughness tester (TALYSURF CCI LITE), with high pass filtering based on the ISO 4287 standard, as shown in Figure 3. The measurement was made in the perpendicular direction to the cutting direction. MCDT was performed to obtain a lower surface roughness at higher MRR to augment the machinability of the tested composite samples using the AWJM process. The taper was evaluated using TA and Equation (1).

(1) $$\tan\theta_{t}=\frac{\frac{p-q}{2}}{h}$$

where p and q are the entry and exit gap distance with the cutting thickness of h.

### 2.3. Taguchi–DEAR Technique

The current study was conducted with four input process control factors and with three levels. Hence, an L_9_ orthogonal table (OA) design was chosen according to the Taguchi technique. Since the present investigation was performed with three performance measures, it was necessary to implement MCDT in the process. In the proposed DEAR approach, the optimal levels of control factors can be computed using a computed ratio, depending on a certain mapping process between a set of original responses and this ratio. This computed ratio is defined as the multi-response-performance-index (MRPI) value, to obtain the optimal arrangement of the control factors. The below mentioned procedures are performed in the proposed approach:

1. Determining the weight (w) value for each response.
2. Computing the weighted data of all trials data.
3. Finding the ratio of MRR weigh to smaller-the-better values.
4. Calculation of MRPI.

(2) $$\mathrm{MRPI}=\frac{R}{S+T}$$

(3) $$R={\mathrm{MRR}*W}_{\mathrm{MRR}}$$

(4) $$S=R_{a}{*\;W}_{R_{a}}$$

(5) $$T={\mathrm{TA}*W}_{\mathrm{TA}}$$

The response weights for all process control variables are computed using the following equations:

(6) $$W_{\mathrm{MRR}}=\frac{\mathrm{MRR}}{\sum \mathrm{MRR}}$$

(7) WRa=1Ra∑1Ra

(8) WTA=1TA∑1TA

## 3. Results and Discussion

In the current work, nine cutting trials were performed according to the proposed experimental design, to examine the effects of AWJM parameters on the machinability of workpiece specimens. Each trial was conducted on three occasions, and the average was considered as the final measured value. The lower standard deviation and standard error values proved the accuracy of the measured values. Table 2 lists the experimental outcomes of all the conducted trials.

### 3.1. Effects of Process Factors on Performance Measures

**Figure 4:**
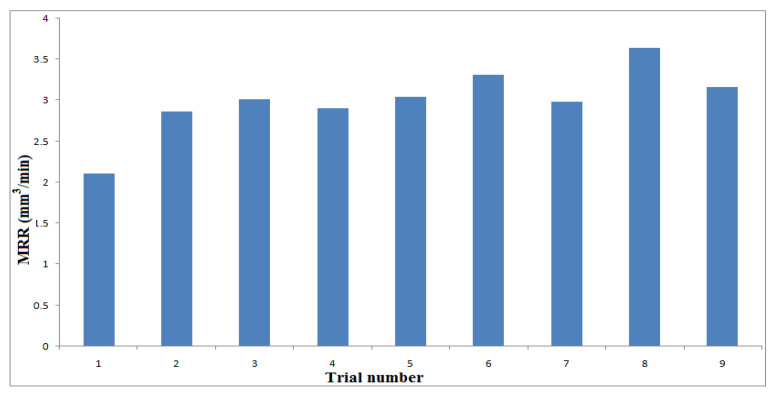
Influence of process parameters on MRR under different trials.

**Figure 5:**
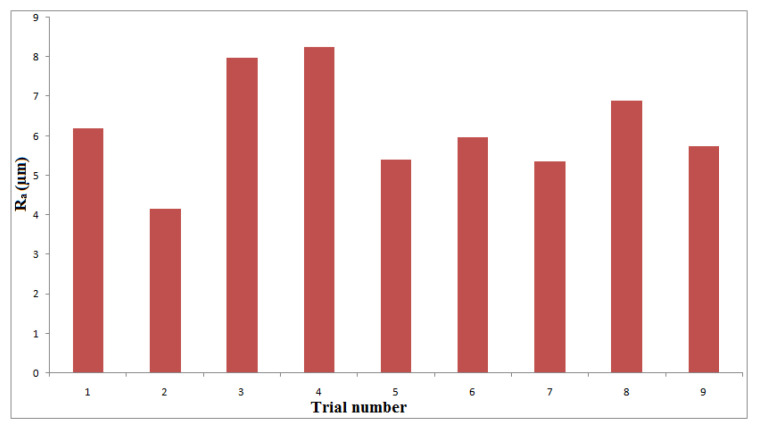
Influence of process parameters on R_a_ under different trials.

**Figure 6:**
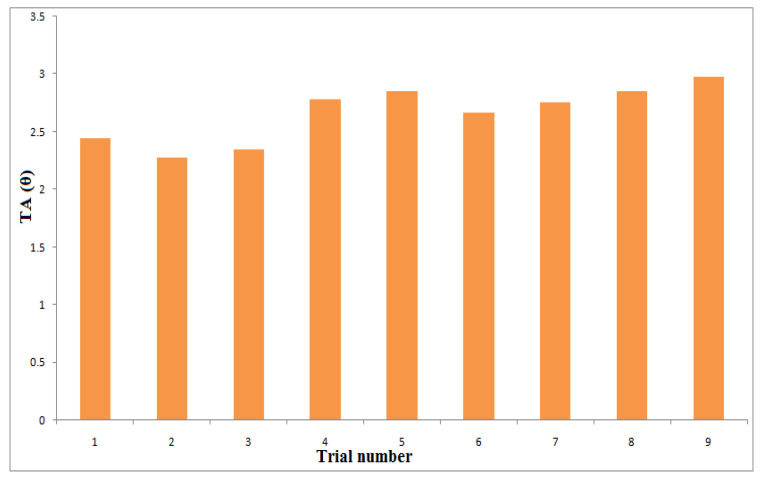
Influence of process parameters on TA under different trials.

**Figure 7:**
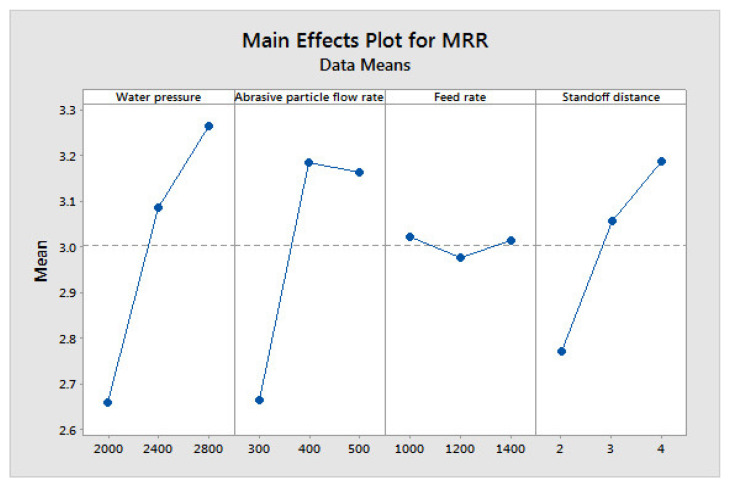
Main effects plot on MRR in AWJM.

**Figure 8:**
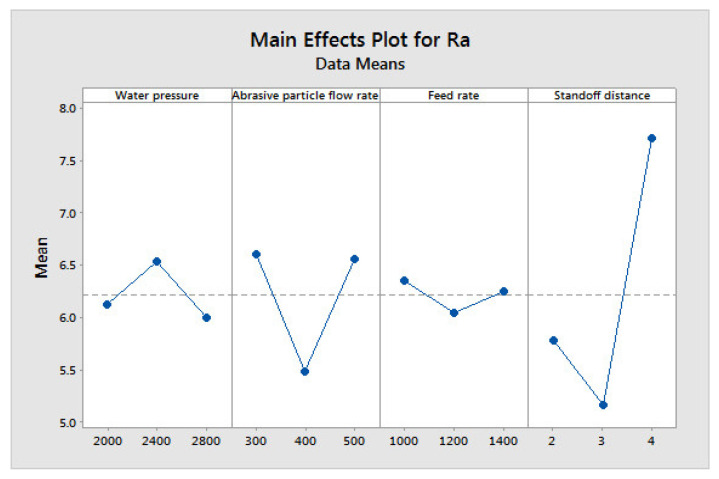
Main effects plot on R_a_in AWJM.

**Figure 9:**
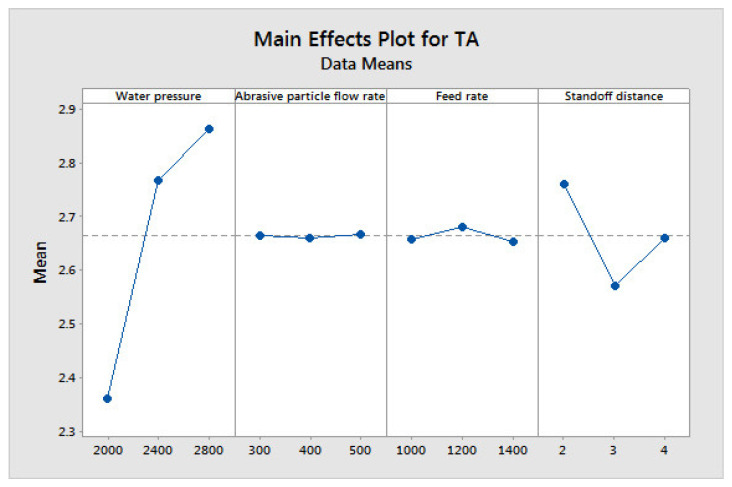
Main effects plot on TA in AWJM.

Figure 4, Figure 5 and Figure 6 show the influences of the process parameters on the quality measures; such as MRR, R_a_, and TA on the AWJM process. A higher water pressure produces a higher MRR, due to the greater impact energy. It was inferred that trial number 8 could remove more material with a higher water pressure and lower feed rate, due to the larger impact energy with a lower feed rate. A lower water pressure can produce tiny craters. It was observed that trial number 2 produced a lower R_a_, owing to the lower WP and moderate standoff distance. It was observed that trial number 2 produced a lower TA, owing to the lower WP with abrasive flow rate. Figure 7, Figure 8 and Figure 9 show the effects of the process parameters on MRR, R_a_, and TA using a main effects plot. The main effects plot was determined using the Minitab 17 software package. The deviation of the response line from the mean line indicates the influence of the process factors on the performance measures. Since the water pressure can increase the impact energy over the machined surface, it possesses a high influence for determining the MRR. The standoff distance with a lower water pressure produces tiny craters. Hence, it has a more influential role in R_a_. Owing to its ability to penetrate into the workpiece specimens with abrasive particles, the WP has a greater influence in determining the TA.

### 3.2. Computation of Significant Process Factors

Table 3 shows the weights of each outcome parameter, along with the MRPI values. The consolidated MRPI of all control variables with their corresponding levels are shown in Table 4; this means that the average value of the all individual performance measures across all the corresponding trials that contain the respective level of variables [14,15]. The highest value of each process parameters specifies the optimal level of input parameters for formulating the outcome measures. From the Table 4, it was found that WP (level 3), AF (level 2), FR (level 1), and SD (level 3) were the optimal arrangement of process factors in the current investigation. MRPI values with different combinations of process control variables for the AWJM process were calculated using a Taguchi–DEAR technique. The maximum value of each control variable indicates the optimal level of that input control variable for any machining process based on different performance measures. It was inferred that the optimal arrangement of process control variables in the process of the current study were 400 mg/min (AF), 4 mm (SOD), 2800 bar (WP), and 1000 mm/min (FR), as shown in Table 5. The highest value of max–min reveals the greatest importance of process control variables on the machining characteristics. It was observed that water pressure has higher significance for determining the quality measures, due to its ability to increase the impact energy on the composite specimens.

**Figure 10:**
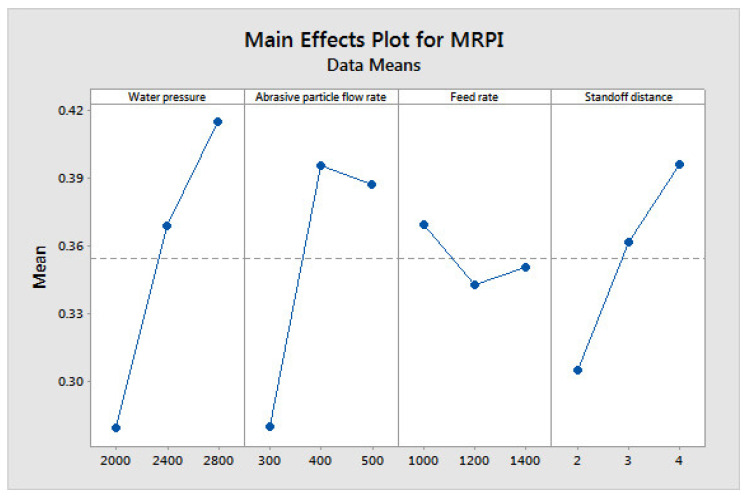
Main effects plot on MRPI in AWJM.

The impact energy of the water is influenced by the gas pressure. This creates larger craters, for producing a higher material removal rate. A higher impact energy is also produced with a lower taper angle. The Ra is also influenced by the crater size. Hence the GP possesses a greater influence on the quality measures in the cutting process. The particles can be ejected from the specimen due to the colloidal of water at very high pressure, along with the SiC particles. The crater size distribution could be determined by the R_a_ in the cutting process. Meanwhile, the crash energy is determined by SOD, it could also contribute to the machinability. Figure 10 shows the influence of process factors on MRPI, using a main effects plot. It was also proven that GP has great effect on the performance measures in the AWJM process.

### 3.3. Computation of Significant Process Factors

A confirmation trial was needed to confirm the conclusions derived from the investigation. It was performed using the optimal levels of the significant factors. The outcome values obtained from the confirmation experiments under the computed settings were 3.575 mm^3^/min (MRR), 4.120 µm (R_a_), and 2.18 degree (TA). The DEAR approach based MRPI value of the confirmation experiment deviated by 0.8% from the mean value. Hence, the accuracy of the prediction was proven.

### 3.4. Surface Analysis under Optimal Control Variables

**Figure 11:**
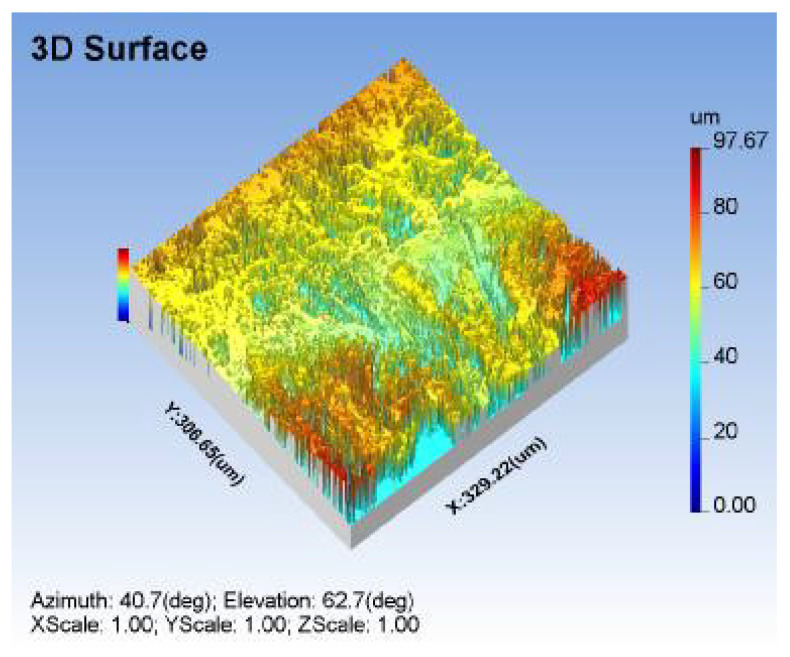
Surface roughness and texture analysis of machined specimen under optimal factors.

**Figure 12:**
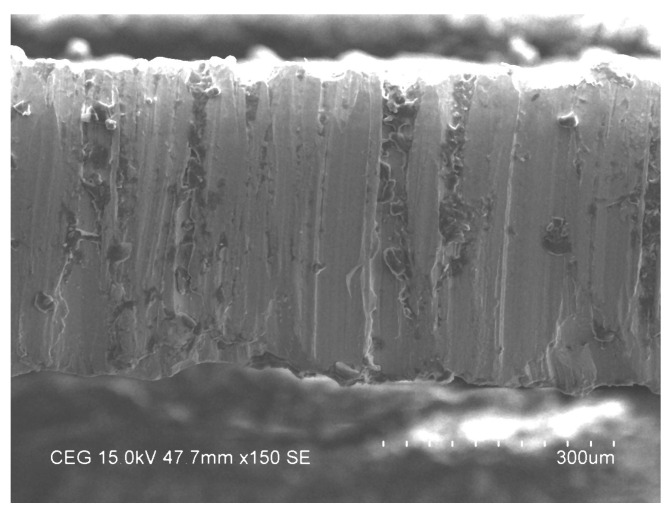
Surface morphology and taper analysis of a specimen under the optimal factors.

The surface topography was investigated under the optimum input factors of AWJM process using surface roughness SEM test, as mentioned in Figure 11 and Figure 12, respectively. Small craters were encountered, with a uniform distribution on the machined surface, due to the optimal gas pressure and standoff distance under the optimal parameter combination. Since it produces tiny craters on the surface, a lower R_a_ was observed in the process, as shown in Figure 11. Across-sectional view of the machined specimen should be investigated for a better surface morphology analysis. The optimal impact energy could also ensure a lower taper over the cutting zone, as shown in Figure 12. The optimal combination enhanced the surface topography, due to the required impact energy during the material removal process.

## 4. Conclusions

In the current investigation, an effort was made to introduce a Taguchi–DEAR methodology in a AWJM process, while machining SiC-reinforced aluminum composite. The following conclusions were drawn:

⮚ The optimal arrangement of input factors in the AWJM process were found to be 2800 bar (WP), 400 mg/min (AF), 1000 mm/min (FR), and 4 mm (SOD), among the elected factors and with the error accuracy of 0.8%.
⮚ The gas pressure is a significant factor for formulating the quality measures, owing to its ability to modify the impact energy and crater size of the machined specimen.
⮚ Since the removal energy can be determined by the standoff distance, this can also contribute to the quality measures.

## Figures and Tables

**Table 1:** Influence of tool electrodes on the ECM process.

Process Parameters	Unit	Variables
WP	bar	2000, 2400, 2800
AF	mg/min	300, 400, 500
SD	mm	2, 3, 4
FR	mm/min	1000, 1200, 1400

**Table 2:** Calculation of performance measures in AWJM.

Input Factors				Quality Measures		
WP	AF	FR	SD	MRR (mm^3^/min)	R_a_ (µm)	TA (θ)
2000	300	1000	2	2.105	6.205	2.45
2000	400	1200	3	2.865	4.165	2.28
2000	500	1400	4	3.012	7.992	2.35
2400	300	1200	4	2.902	8.252	2.78
2400	400	1400	2	3.042	5.402	2.85
2400	500	1000	3	3.315	5.965	2.67
2800	300	1400	3	2.988	5.368	2.76
2800	400	1000	4	3.645	6.895	2.85
2800	500	1200	2	3.165	5.735	2.98
Mean (M)				3.004	6.219	2.663
Standard deviation (SD)				0.414	1.306	0.246
Standard error (SE)				0.138	0.435	0.082

**Table 3:** Calculation of weights and MRPI.

Trial No.	Weights			MRPI
	MRR	R_a_ (µm)	TA (θ)	
1.	0.077851	0.107011	0.11983	0.171133
2.	0.105958	0.159425	0.128765	0.317015
3.	0.111395	0.083084	0.124929	0.350381
4.	0.107326	0.080466	0.105606	0.325256
5.	0.112504	0.122918	0.103012	0.357395
6.	0.122601	0.111317	0.109957	0.424422
7.	0.110507	0.123697	0.106371	0.344819
8.	0.134805	0.096302	0.103012	0.513128
9.	0.117053	0.115781	0.098518	0.386882

**Table 4:** Computation of average MRPI values.

Factors	Levels			Max–Min
	1	2	3	
WP	0.27951	0.36902	0.41494	0.13543
AF	0.28040	0.39585	0.38723	0.11544
FR	0.36956	0.34305	0.35087	0.02651
SD	0.30514	0.36209	0.39626	0.09112

**Table 5:** Identification of optimum factors.

Factors	Variable	Unit
Water pressure	2800	bar
Abrasive particle flow rate	400	mg/min
Feed rate	1000	mm/min
Standoff distance	4	mm
